# CircSAMD4A contributes to cell doxorubicin resistance in osteosarcoma by regulating the miR-218-5p/KLF8 axis

**DOI:** 10.1515/biol-2020-0079

**Published:** 2020-11-24

**Authors:** Wei Wei, Liefeng Ji, Wanli Duan, Jiang Zhu

**Affiliations:** Department of orthopedics, Shaoxing Shangyu People’s Hospital, No. 517, Shimin Avenue, Baiguan Street, Shangyu District, Shaoxing, Zhejiang Province, 312300, China

**Keywords:** circSAMD4A, doxorubicin resistance, osteosarcoma, miR-218-5p, KLF8

## Abstract

Circular RNA sterile alpha motif domain containing 4A (circSAMD4A) was found to be differentially expressed in osteosarcoma and contributed to the tumorigenesis of osteosarcoma. However, the role of circSAMD4A in doxorubicin (DXR) resistance of osteosarcoma is yet to be elucidated. Levels of circSAMD4A, microRNA (miR)-218-5p and Krüppel-like factor 8 (KLF8) were detected using quantitative reverse transcription-polymerase chain reaction. Western blot was applied to detect the protein levels of KLF8, cyclin D1 and p21. Cell viability, cell cycle, migration and invasion were analyzed using Cell Counting Kit-8 assay, flow cytometry and transwell assay, respectively. The interaction between miR-218-5p and circSAMD4A or KLF8 was verified using dual-luciferase reporter assay or RNA immunoprecipitation assay. *In vivo* experiments were performed using murine xenograft models. CircSAMD4A and KLF8 were elevated in osteosarcoma, and knockdown of circSAMD4A or KLF8 sensitized osteosarcoma cells to DXR by mediating resistant cell viability, migration and invasion inhibition, and cell cycle arrest *in vitro*. miR-218-5p was decreased in osteosarcoma, and miR-218-5p inhibition enhanced DXR resistance. Besides, miR-218-5p was found to bind to circSAMD4A or KLF8, and subsequent rescue experiments indicated that miR-218-5p inhibition reversed the inhibitory effects of circSAMD4A silencing on DXR resistance, and silencing miR-218-5p enhanced DXR resistance by targeting KLF8 in osteosarcoma cells. Moreover, circSAMD4A could indirectly regulate KLF8 via miR-218-5p. Additionally, circSAMD4A knockdown enhanced the cytotoxicity of DXR in osteosarcoma *in vivo* via regulating miR-218-5p and KLF8. In all, circSAMD4A enhanced cell DXR resistance in osteosarcoma by regulating the miR-218-5p/KLF8 axis, suggesting a novel therapeutic target for therapy-resistant osteosarcoma.

## 1 Introduction

Osteosarcoma is the most frequent primary solid malignancy of bone, with a higher incidence in children and adolescents [1]. Standardized application of neoadjuvant chemotherapy plays key roles in the treatment of osteosarcoma, which significantly improves limb salvage and survival rates [2]. Doxorubicin (DXR) is one of the most active drugs for osteosarcoma treatment; however, DXR resistance gradually emerged in osteosarcoma patients, which limits the effects of the drug [3]. Thus, further investigations on the molecular mechanisms of DXR resistance are necessary to develop new targets to prevent DXR resistance.

Recent studies have suggested that non-coding RNAs (ncRNAs) and ncRNA-regulatory processes are involved in drug resistance in multiple types of cancers [4]. The ncRNAs account for greater than 90% of human RNAs and cannot encode proteins [5]. It has been documented that ncRNAs function as underlying players in multiple cellular processes, including cell cycle, differentiation, proliferation, metastasis, angiogenesis and oxidative stress [6,7,8]. Circular RNAs (circRNAs) are a new class of highly conserved ncRNAs forming a covalently closed continuous loop. Emerging evidence has identified the association between circRNAs and drug resistance in osteosarcoma [9,10]. Circular RNA sterile alpha motif domain containing 4A (circSAMD4A) is a newly identified circRNA, and Yanbin et al. found that circSAMD4A enhanced cell proliferation and the features of cancer stem cells in osteosarcoma by upregulating miR-1244-mediated MDM2 expression, suggesting the carcinogenic role of circSAMD4A in osteosarcoma [11]. However, the function of circSAMD4A in drug resistance in osteosarcoma remains unclear.

MicroRNAs (miRNAs) are well-documented small ncRNAs of approximately 22 nucleotides in length, which control specific gene expression programs by the regulation of post-transcriptional processes [12]. MiRNAs have been investigated to have vital functions in a variety of physiological and pathobiological processes, such as tumorigenesis and angiogenesis [13]. Besides, miRNAs also mediate chemoresistance of osteosarcoma, offering a new therapeutic target for osteosarcoma [14]. MiR-218-5p is a well-recognized tumor suppressor in various cancers [15,16], while the function of miR-218-5p in osteosarcoma is yet to be elucidated. Krüppel-like factor 8 (KLF8) is a protein encoded by KLF8 gene, which is a member of the KLF protein family. KLF8 has a significant role in regulating oncogenic transformation, cell cycle and epithelial to mesenchymal transition [17,18,19]. Recently, KLF8 was found to promote osteosarcoma carcinogenesis and progression [20,21]. Thus, we hypothesized that KLF8 might be associated with osteosarcoma chemoresistance.

In this study, we attempted to detect the functions of circSAMD4A, miR-218-5p and KLF8 in DXR resistance in osteosarcoma and explored whether there was a potential regulatory network among circSAMD4A, miR-218-5p and KLF8.

## 2 Materials and methods

### 2.1 Clinical samples

Tumor tissues and para-carcinoma tissues from 60 osteosarcoma patients who underwent surgical resection at Shaoxing Shangyu People’s Hospital were obtained and immediately stored at −80°C until use. All patients were treated preoperatively with DXR-based chemotherapy and were divided into the DXR-resistant group (treatment-resistant, *N* = 36) and the DXR-sensitive group (treatment-responsive, *N* = 24) depending on the sensitivity of osteosarcoma patients to DXR.


**Informed consent:** Informed consent has been obtained from all individuals included in this study.
**Ethical approval:** The research related to human use has been complied with all the relevant national regulations, institutional policies and in accordance with the tenets of the Helsinki Declaration and has been approved by the Ethics Committee of Shaoxing Shangyu People’s Hospital.

### 2.2 Cell culture

Human osteosarcoma cell lines HOS and U2OS and human osteoblast cell line hFOB1.19 were obtained from the Shanghai Academy of Life Science (Shanghai, China). HOS and U2OS cells were cultured in McCoy’s 5A medium (Gibco, Los Angeles, CA, USA) supplemented with 10% fetal bovine serum (FBS; Gibco) and ampicillin and streptomycin. hFOB cells were grown in Dulbecco’s modified Eagle medium/F12 containing 10% FBS. All cells were incubated with 5% CO_2_ at 37°C.

DXR-resistant HOS (HOS/DXR) and U2OS (U2OS/DXR) cells were generated by continuously exposing parental HOS and U2OS cells to stepwise increasing doses of DXR (Sigma, San Francisco, CA, USA) over several months. DXR-resistant cells were cultured in the same media containing 1 µg/mL DXR at 37°C with 5% CO_2_ to retain their drug-resistant phenotype.

### 2.3 Quantitative reverse transcription-polymerase chain reaction (qRT-PCR)

TRIzol reagent (Invitrogen, Carlsbad, CA, USA) was used to conduct the extraction of total RNA by following the standard procedure. The synthesis of complementary DNAs (cDNAs) was performed using the PrimeScript RT reagent kit (Takara, Dalian, China), and then the synthesized cDNA template was amplified with SYBR Green I (Takara) on ABI7300. Fold changes were calculated by the 2^−ΔΔCt^ method using glyceraldehyde-3-phosphate dehydrogenase (GAPDH) or U6 small nuclear RNA (U6) as the normalization control. The primers used were as follows: circSAMD4A: F 5′-TGAAGCCAGGAAACCTCGAC-3′, R 5′-GCCAGTCCTAGACCCAGGTA-3′; miR-218-5p: F 5′-AGCGAGATTTTCTGTTGTGCTT-3′, R 5′-GACGTTCCATGGTGCTTGAC-3′; KLF8: F 5′-GCTCACCGCAGAATCCATACA-3′, R 5′-GTGCACCGAAAAGGCTTGAT-3′; GAPDH: F 5′-CCCACATGGCCTCCAAGGAGTA-3′, R 5′-GTGTACATGGCAACTGTGAGGAGG-3′; U6: F 5′-GCTTCGGCAGCACATATACTAA-3′, R 5′-AACGCTTCACGAATTTGCGT-3′.

### 2.4 Cell transfection

The mimic and inhibitor of miR-218-5p (miR-218-5p mimic and anti-miR-218-5p) and their controls (miR-NC mimic and anti-NC) were obtained from RiboBio (Guangzhou, China). Small interfering RNA (siRNA) oligonucleotides targeting circSAMD4A (si-circSAMD4A; siRNA: 5′-AGCACAAGTACAAGAGGAAATdTdT-3′), siRNA oligonucleotides targeting KLF8 (si-KLF8; siRNA: 5′-UGAGUUUAUCCAUAUCGACCA-3′), siRNA oligonucleotides (si-NC), the scramble short hairpin RNA (shRNA) sequence (sh-NC) and shRNA targeting circSAMD4A (sh-circSAMD4A) were synthesized by Invitrogen. The transfection was conducted using Lipofectamine™ 2000 (Invitrogen) by following the instructions of the manufacturer.

### 2.5 Cell viability assay

Resistant cells transfected with the assigned vector for 48 h were seeded in 96-well plates (5,000 cells/well) overnight, and then they were exposed to increasing concentrations of DXR (0, 0.5, 1, 2, 4, 8 or 16 µg/mL), followed by incubation for another 48 h. Afterward, each well was incubated with Cell Counting Kit-8 (CCK-8) solution (10 µL/well; Beyotime, Shanghai, China) for about 2 h. Subsequently, the optical density was measured at 450 nm using a microplate reader, and the half-maximal inhibitory concentration (IC_50_) value was calculated for each cell line.

### 2.6 Cell cycle analysis

The transfected cells were harvested, and then the cells (1 × 10^5^) were digested using trypsin to collect single-cell suspensions. After that, the cells were fixed with 75% ethanol for 4 h at 4°C, followed by incubation with propidium iodide (Cell Cycle Detection kit; BD Biosciences, San Jose, CA, USA). The percentage of cells in the G0/G1, S or G2/M phase was measured by flow cytometry with a FACS Calibur system (BD Bioscience).

### 2.7 Western blot

The extracted proteins were separated by sodium dodecyl sulfate-polyacrylamide gel electrophoresis and electrophoretically transferred to polyvinylidene difluoride membranes (Millipore, Billerica, MA, USA), and then the membranes were incubated with primary antibodies against cyclin D1 (1:20,000; ab134175, Abcam, Cambridge, MA, USA), p21 (1:3,000; ab188224, Abcam), KLF8 (1:5,000; ab168527, Abcam) and horseradish peroxidase-conjugated secondary antibody (1:1,000; Sangon, Shanghai, China). Immunoreactive bands were visualized using an enhanced chemiluminescence kit (Beyotime) and normalized using GAPDH (1:10,000; ab8245, Abcam).

### 2.8 Cell migration and invasion analysis

Transwell chambers pre-coated with Matrigel (BD Biosciences) or uncoated were used to determine cell invasion or migration, respectively. The cells transfected with the assigned vector for 48 h were placed in the upper chambers with 200 µL of serum-free McCoy’s 5A medium, and the lower chambers were filled with 500 µL of McCoy’s 5A medium with FBS. After 24 h, the cells on the lower face of the membranes were fixed and stained, and counted using a microscope in five different fields.

### 2.9 Dual-luciferase reporter assay

MiR-218-5p in circSAMD4A or KLF8 3′-UTR wild-type (WT) and their mutated (MUT) sequences were separately cloned into pRL-TK luciferase plasmids (Promega, Shanghai, China). Then HOS/DXR and U2OS/DXR cells were co-transfected with 100 ng of constructed luciferase reporter plasmid and 40 nM miR-218-5p or miR-NC using Lipofectamine™ 2000 (Invitrogen). Finally, luciferase activity was detected using a dual luciferase assay kit (Promega).

### 2.10 RNA immunoprecipitation (RIP) assay

Resistant cells transfected with miR-218-5p mimic or miR-NC mimic were lysed using RIPA buffer, and then 100 µL of cell lysates was incubated with RIPA buffer containing magnetic beads conjugated with human anti-Argonaute2 (Ago2) antibody (Millipore) or normal mouse IgG (Millipore), followed by interaction with Proteinase K to digest the protein. Subsequently, immunoprecipitated RNA was extracted, and purified RNA was subjected to qRT-PCR analysis to examine the expression of circSAMD4A.

### 2.11 *In vivo* chemosensitivity assay

BALB/c nude mice (male, aged 3–5 weeks, *N* = 12) purchased from the National Laboratory Animal Center (Beijing, China) were divided into four groups with three mice in each group to establish mouse models. First, each mouse of two groups was subcutaneously injected with U2OS/DXR cells transfected with lentivirus-(lenti)-sh-NC, followed by treatment with PBS or DXR (3 mg/kg) every 3 days after 1 week of inoculation. Also, U2OS/DXR cells transfected with lenti-sh-circSAMD4A (sh-circSAMD4A) were subcutaneously injected into each mouse from the other two groups, followed by treatment with PBS or DXR (3 mg/kg) every 3 days after 1 week of inoculation. The volume of the tumor was calculated every week. At day 28, mice were killed, and tumor masses were weighed and collected for further molecular analysis.


**Ethical approval:** The research related to animal use has been complied with all the relevant national regulations and institutional policies for the care and use of animals and has been approved by the Animal Research Committee of Shaoxing Shangyu People’s Hospital and implemented in line with the guidelines of the National Animal Care and Ethics Institution.

### 2.12 Statistical analysis

Data from thrice-repeated experiments were expressed as mean ± standard deviation and analyzed using GraphPad Prism 7 software. Statistical difference was detected using Student’s *t*-test or one-way analysis of variance followed by the Tukey *post hoc* test in different groups. *P* <  0.05 indicated statistical significance.

## 3 Results

### 3.1 Expression of circSAMD4A, miR-218-5p and KLF8 in DXR-resistant osteosarcoma tissues and cell lines

**Figure 1:**
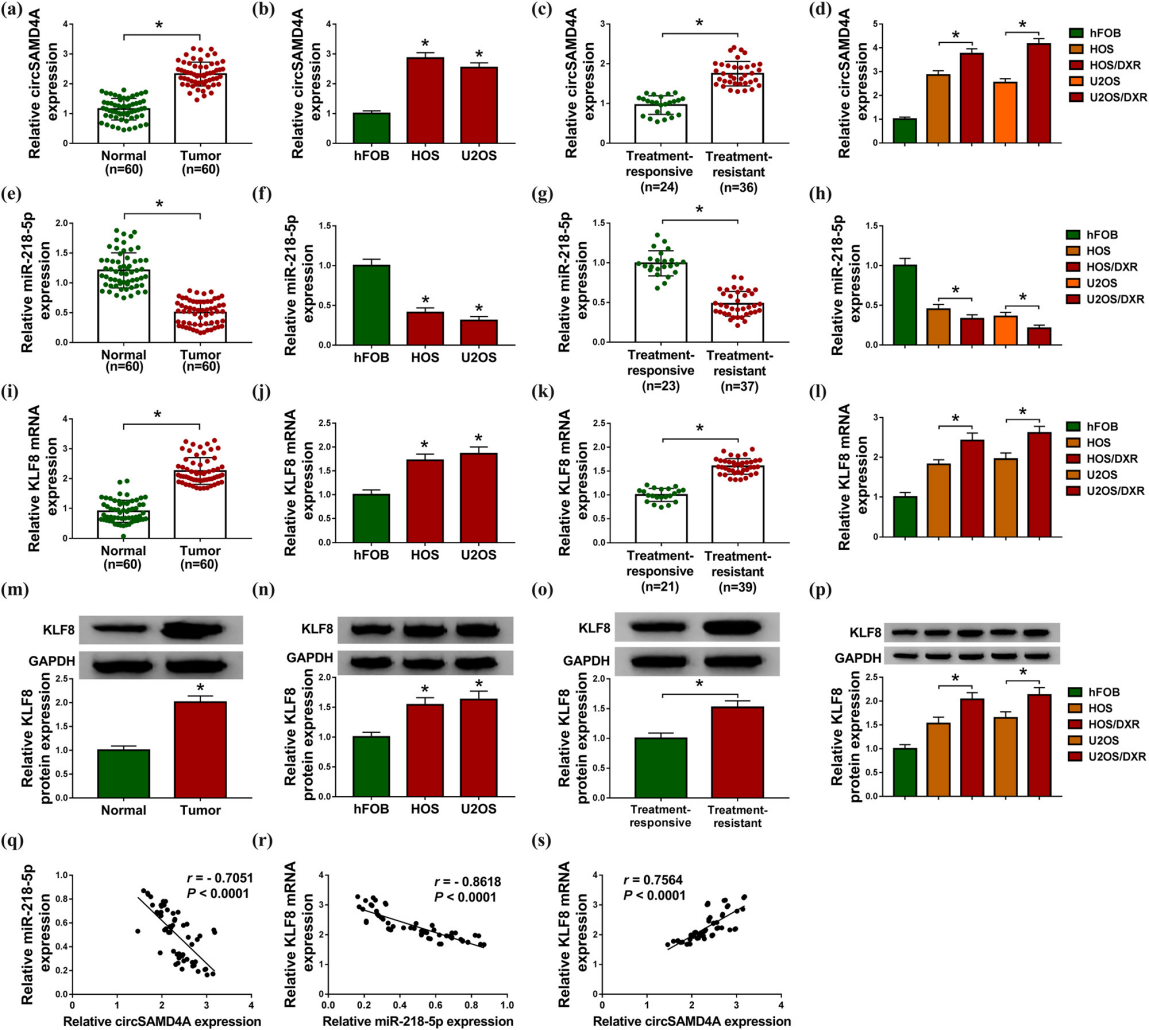
The expression of circSAMD4A, miR-218-5p and KLF8 in DXR-resistant osteosarcoma tissues and cell lines. (a–p) Analysis of circSAMD4A, miR-218-5p and KLF8 expression levels in osteosarcoma tissues and matched non-tumor tissues (a, e, i and m), osteosarcoma cell lines (HOS and U2OS) and normal cell line hFOB (b, f, j and n), treatment-resistant group and treatment-responsive group (c, g, k and o) and DXR-resistant cell lines HOS/DXR and U2OS/DXR and their parental HOS and U2OS cells (d, h, l and p) using qRT-PCR or western blot. Each experiment was repeated three times, and the average was taken. (q–s) Correlation analysis between miR-218-5p and circSAMD4A or KLF8. *P < 0.05.

The levels of circSAMD4A, miR-218-5p and KLF8 were detected, and the results showed that relative to the non-tumor tissues and normal cell line hFOB, circSAMD4A (Figure 1a and b) and KLF8 (Figure 1i, j, m and n) were elevated, while miR-218-5p (Figure 1e and f) was decreased in osteosarcoma tissues and cell lines (HOS and U2OS). Importantly, osteosarcoma tissues were divided into the DXR-resistant group (treatment-resistant, *N* = 36) and the DXR-sensitive group (treatment-responsive, *N* = 24) depending on the sensitivity of osteosarcoma patients to DXR, and we found that circSAMD4A (Figure 1c) and KLF8 (Figure 1k and o) were notably higher, while miR-218-5p (Figure 1g) was lower in the treatment-resistant group than those in the treatment-responsive group. Similarly, in contrast with the parental osteosarcoma cell lines HOS and U2OS, circSAMD4A (Figure 1d) and KLF8 (Figure 1l and p) were also significantly increased and miR-218-5p (Figure 1h) was decreased in DXR-resistant cell lines HOS/DXR and U2OS/DXR. Additionally, we also discovered that miR-218-5p expression was negatively correlated with circSAMD4A (*r* = −0.7051, *P* < 0.0001; Figure 1q) and KLF8 (*r* = −0.8618, *P* < 0.0001; Figure 1r), and KLF8 expression was positively correlated with circSAMD4A in osteosarcoma tissues (*r* = 0.7564, *P* < 0.0001; Figure 1s). These data indicated that the dysregulation of circSAMD4A, miR-218-5p or KLF8 was associated with the DXR resistance, and there might be a connection among them in osteosarcoma.

### 3.2 CircSAMD4A knockdown mitigates DXR resistance in osteosarcoma *in vitro*

**Figure 2:**
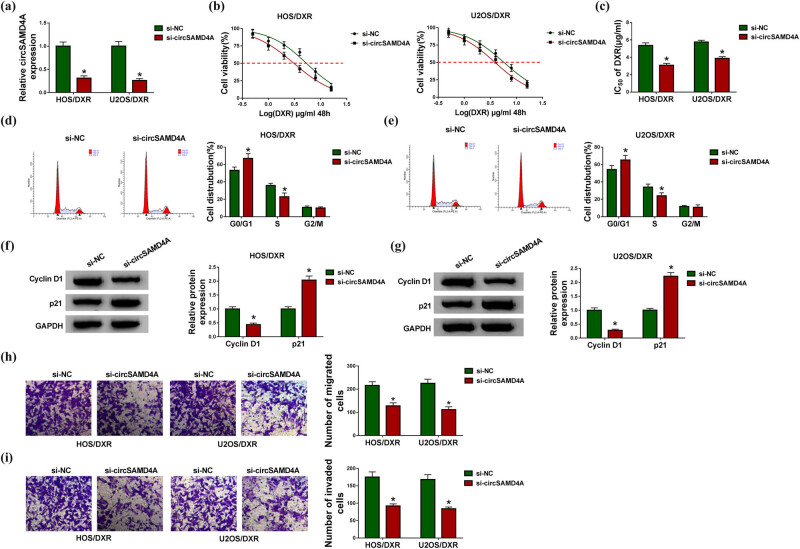
CircSAMD4A knockdown mitigates DXR resistance in osteosarcoma *in vitro.* HOS/DXR and U2OS/DXR cells were transfected with si-NC or si-circSAMD4A. (a) qRT-PCR analysis of circSAMD4A expression in HOS/DXR and U2OS/DXR cells. (b) CCK-8 assay of resistant cell viability with increasing concentrations of DXR (0, 0.5, 1, 2, 4, 8 or 16 µg/mL). (c) CCK-8 assay of the IC_50_ values of resistant cells to DXR. (d and e) Flow cytometry analysis of the cell cycle of resistant cells. (f and g) Western blot analysis of cyclin D1 and p21 levels in resistant cells. (h and i) Transwell assay of the migration and invasion abilities of resistant cells. The experiments were performed three times. *P < 0.05.

The function of circSAMD4A in DXR resistance in osteosarcoma cells was analyzed in detail. HOS/DXR and U2OS/DXR cells were transfected with si-NC or si-circSAMD4A, and qRT-PCR analysis showed that si-circSAMD4A transfection significantly reduced the level of circSAMD4A in cells relative to the si-NC transfection (Figure 2a). Subsequently, CCK-8 assay exhibited that circSAMD4A knockdown combined with increasing doses of DXR (0, 0.5, 1, 2, 4, 8 or 16 µg/mL) gradually inhibited the viability of HOS/DXR and U2OS/DXR cells (Figure 2b); besides that, the IC_50_ values of HOS/DXR and U2OS/DXR cells for DXR in the circSAMD4A knockdown group were markedly lower than those in the cells of the si-NC group (Figure 2c). Meanwhile, we found that the number of HOS/DXR and U2OS/DXR cells in the S phase was decreased upon circSAMD4A silencing, while cells in the G0/G1 phase were accumulated, indicating cell cycle arrest (Figure 2d and e); also, the downregulation of cyclin D1 levels and upregulation of p21 levels induced by circSAMD4A knockdown in HOS/DXR and U2OS/DXR cells further suggested the cell cycle arrest (Figure 2f and g). In addition, transwell assay showed that the number of migrated and invaded HOS/DXR and U2OS/DXR cells was declined by circSAMD4A downregulation (Figure 2h and i). Taken together, circSAMD4A knockdown sensitized osteosarcoma cells to DXR by inhibiting resistant cell viability, cell cycle progression, migration and invasion.

### 3.3 CircSAMD4A is a sponge of miR-218-5p

**Figure 3:**
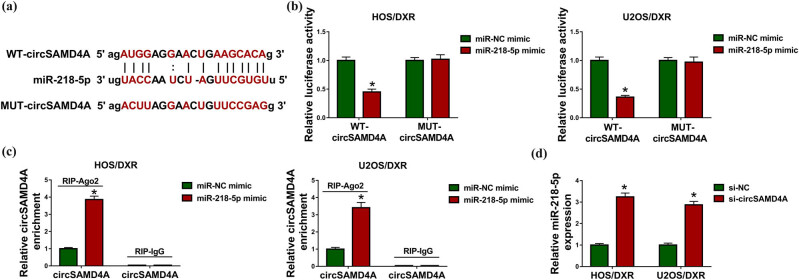
CircSAMD4A is a sponge of miR-218-5p. (a) The potential binding sites of circSAMD4A and miR-218-5p. (b) Dual-luciferase reporter assay in HOS/DXR and U2OS/DXR cells co-transfected with the reporter plasmid and the indicated miRNAs. (c) RIP analysis for the enrichment of Ago2 on circSAMD4A in HOS/DXR and U2OS/DXR cells. (d) qRT-PCR analysis of miR-218-5p expression in HOS/DXR and U2OS/DXR cells transfected with si-NC or si-circSAMD4A. The experiments were performed three times. *P < 0.05.

Based on the above results, we knew that miR-218-5p expression was negatively correlated with circSAMD4A; thus, the potential relationship between them was investigated. According to the prediction of the StarBase program, we found that miR-218-5p might be a target of circSAMD4A (Figure 3a). To verify this prediction, a dual luciferase reporter assay was performed, and the results displayed that miR-218-5p overexpression significantly reduced the luciferase activity in HOS/DXR and U2OS/DXR cells transfected with WT-circSAMD4A, and there was no obvious change in cells transfected with MUT-circSAMD4A (Figure 3b). Additionally, RIP assay demonstrated that miR-218-5p upregulation elevated the enrichment of Ago2 on circSAMD4A both in HOS/DXR and U2OS/DXR cells (Figure 3c). Interestingly, we observed that circSAMD4A knockdown increased miR-218-5p expression in HOS/DXR and U2OS/DXR cells (Figure 3d). Collectively, circSAMD4A directly bound to miR-218-5p and negatively regulated its expression.

### 3.4 CircSAMD4A knockdown sensitizes osteosarcoma cells to DXR by binding to miR-218-5p

**Figure 4:**
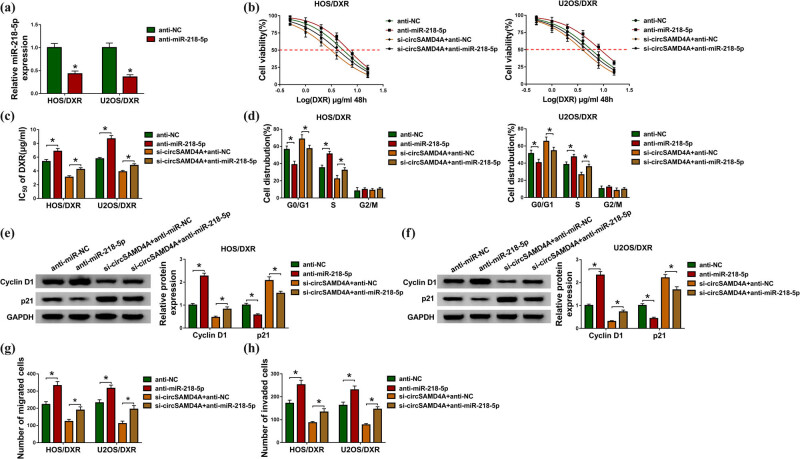
CircSAMD4A knockdown sensitizes osteosarcoma cells to DXR by binding to miR-218-5p. (a) qRT-PCR analysis of miR-218-5p expression in HOS/DXR and U2OS/DXR cells transfected with anti-miR-218-5p or anti-NC. (b–h) HOS/DXR and U2OS/DXR cells were transfected with anti-NC, anti-miR-218-5p, si-circSAMD4A + anti-NC or si-circSAMD4A + anti-miR-218-5p. (b) CCK-8 assay of resistant cell viability with increasing concentrations of DXR (0, 0.5, 1, 2, 4, 8 or 16 µg/mL). (c) CCK-8 assay of the IC_50_ values of HOS/DXR and U2OS/DXR cells to DXR. (d) Cell cycle analysis of resistant cells using flow cytometry. (e and f) Western blot analysis of cyclin D1 and p21 levels in resistant cells. (g and h) Migration and invasion analyses of resistant cells with transwell assay. All experiments were repeated three times independently. *P < 0.05.

Whether miR-218-5p is involved in the action of circSAMD4A in DXR resistance in osteosarcoma cells was explored. First, HOS/DXR and U2OS/DXR cells were transfected with anti-miR-218-5p or anti-NC, and we found that miR-218-5p was significantly reduced by anti-miR-218-5p transfection compared to the anti-NC transfection (Figure 4a). After that, we found that miR-218-5p inhibition increased HOS/DXR and U2OS/DXR cell viability with 0.5–16 µg/mL DXR (Figure 4b) and upregulated the values of IC_50_ (Figure 4c). Flow cytometry analysis showed that miR-218-5p inhibition induced DXR-resistant cell cycle progression, reflected by the reduction of HOS/DXR and U2OS/DXR cells in the G0/G1 phase and elevation in the S phase (Figure 4d), as well as the increase of cyclin D1 and decrease of p21 in HOS/DXR and U2OS/DXR cells (Figure 4e and f). Meanwhile, the number of migrated and invaded HOS/DXR and U2OS/DXR cells was also increased by miR-218-5p inhibition (Figure 4g and h). Thus, all these results indicated that silencing of miR-218-5p promoted DXR resistance in osteosarcoma cells. Next, si-circSAMD4A + anti-NC or si-circSAMD4A + anti-miR-218-5p was transfected into HOS/DXR and U2OS/DXR cells. The results indicated that miR-218-5p inhibition reversed circSAMD4A knockdown-induced HOS/DXR and U2OS/DXR cell viability inhibition (Figure 4b and c), cell cycle arrest (Figure 4d–f), as well as migration and invasion suppression (Figure 4g and h). Altogether, circSAMD4A knockdown sensitized osteosarcoma cells to DXR by positively regulating miR-218-5p expression.

### 3.5 KLF8 is a target of miR-218-5p

**Figure 5:**
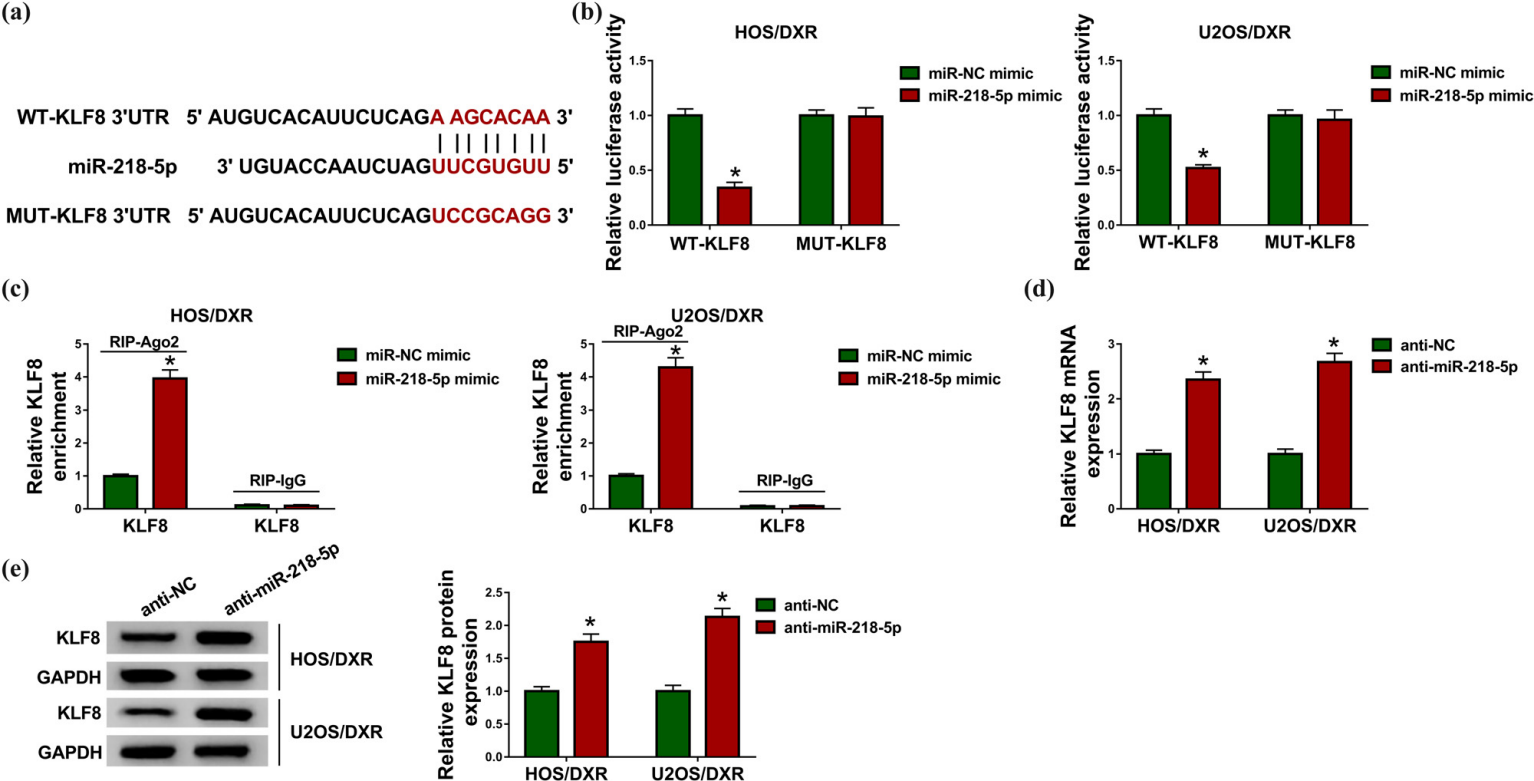
KLF8 is a target of miR-218-5p. (a) Schematic representation of the predicted binding sites of miR-218-5p on KLF8. (b) Dual-luciferase reporter assay in HOS/DXR and U2OS/DXR cells co-transfected with the reporter plasmid and the indicated miRNAs. (c) RIP analysis for the enrichment of Ago2 on KLF8 in HOS/DXR and U2OS/DXR cells. (d and e) Analysis of KLF8 expression level in HOS/DXR and U2OS/DXR cells transfected with anti-NC or anti-miR-218-5p with qRT-PCR or western blot. The results are presented as the average of three independent experiments. *P < 0.05.

Considering the negative correlation between KLF8 and miR-218-5p, the regulatory relationship between them was evaluated. Through searching the TargetScan program, the putative binding sites of miR-218-5p on KLF8 were predicted (Figure 5a). Then a reduction of luciferase activity in HOS/DXR and U2OS/DXR cells co-transfected with WT-KLF8 and miR-218-5p mimic confirmed the direct interaction between KLF8 and miR-218-5p (Figure 5b). Also, RIP assay suggested that miR-218-5p overexpression increased the enrichment of Ago2 on KLF8 both in HOS/DXR and U2OS/DXR cells, further verifying that miR-218-5p targeted KLF8 (Figure 5c). Subsequently, we found that miR-218-5p inhibition upregulated the expression of KLF8 both at mRNA and protein levels (Figure 5d and e). Therefore, miR-218-5p targetedly repressed KLF8 expression.

### 3.6 KLF8 knockdown attenuates the action of miR-218-5p in DXR resistance in osteosarcoma cells

**Figure 6:**
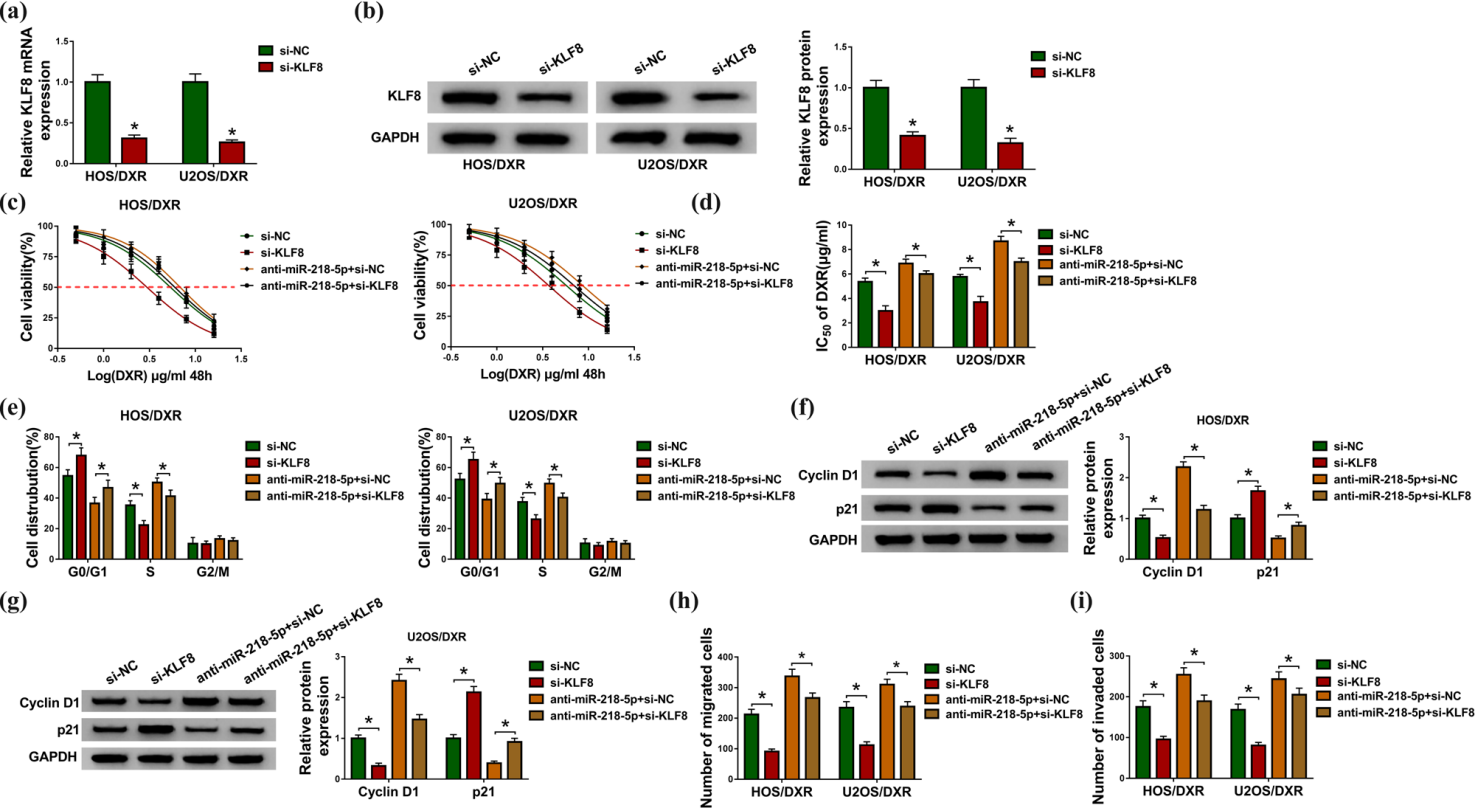
KLF8 knockdown suppresses the action of miR-218-5p in DXR resistance in osteosarcoma cells. (a and b) Analysis of KLF8 expression level in HOS/DXR and U2OS/DXR cells transfected with si-NC or si-KLF8 using qRT-PCR or western blot. (c–i) HOS/DXR and U2OS/DXR cells were transfected with si-NC, si-KLF8, anti-miR-218-5p + si-NC or anti-miR-218-5p + si-KLF8. (c) CCK-8 assay of resistant cell viability with increasing concentrations of DXR (0, 0.5, 1, 2, 4, 8 or 16 µg/mL). (d) CCK-8 assay of the IC_50_ values of resistant cells to DXR. (e) Flow cytometry analysis of the cell cycle of resistant cells. (f and g) Western blot analysis of cyclin D1 and p21 levels in resistant cells. (h and i) Measurement of the number of migrated and invaded resistant cells with transwell assay. All experiments were repeated three times independently. *P < 0.05.

We then studied whether the action of miR-218-5p in DXR resistance in osteosarcoma cells was mediated by KLF8. First, HOS/DXR and U2OS/DXR cells were transfected with si-KLF8 or si-NC, and KLF8 expression was notably reduced by si-KLF8 transfection as expected (Figure 6a and b). After that, CCK-8 assay showed that KLF8 knockdown combined with increasing doses of DXR (0, 0.5, 1, 2, 4, 8 or 16 µg/mL) suppressed HOS/DXR and U2OS/DXR cell viability (Figure 6c), and the IC_50_ values were also reduced by KLF8 silencing in HOS/DXR and U2OS/DXR cells (Figure 6d). In addition, KLF8 knockdown elevated the number of HOS/DXR and U2OS/DXR cells in the G0/G1 phase and reduced cells in the S phase, thereby inducing cell cycle arrest at G0/G1 (Figure 6e); moreover, the upregulation of p21 levels and downregulation of cyclin D1 levels induced by KLF8 knockdown also demonstrated the cell cycle arrest in HOS/DXR and U2OS/DXR cells (Figure 6f and g). Afterward, we found that KLF8 knockdown reduced the number of migrated and invaded HOS/DXR and U2OS/DXR cells (Figure 6h and i). Thus, KLF8 knockdown suppressed DXR resistance in osteosarcoma cells. Next, HOS/DXR and U2OS/DXR cells were transfected with anti-miR-218-5p + si-NC or anti-miR-218-5p + si-KLF8, and we discovered that KLF8 knockdown attenuated miR-218-5p inhibition-mediated DXR-resistant cell viability promotion (Figure 6c and d), cell cycle progression (Figure 6e–g) and cell migration and invasion enhancement (Figure 6h and i) in HOS/DXR and U2OS/DXR cells. These data suggested that miR-218-5p inhibition blocked the sensitivity of osteosarcoma cells to DXR by regulating KLF8.

### 3.7 CircSAMD4A positively regulates KLF8 via miR-218-5p

**Figure 7:**
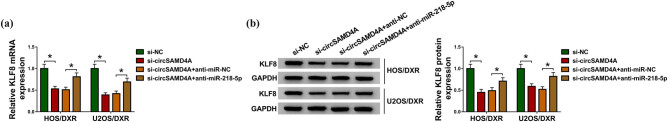
CircSAMD4A positively regulates KLF8 via miR-218-5p. (a and b) Analysis of KLF8 expression level in HOS/DXR and U2OS/DXR cells transfected with si-NC, si-circSAMD4A, si-circSAMD4A + anti-NC or si-circSAMD4A + anti-miR-218-5p using qRT-PCR or western blot. Each experiment was repeated three times, and the average was taken. *P < 0.05.

Given that KLF8 was positively correlated with circSAMD4A in osteosarcoma, we wanted to know whether circSAMD4A regulated KLF8 via miR-218-5p. Then we found that circSAMD4A knockdown reduced the expression level of KLF8 both at mRNA and protein levels, while this reduction was rescued following miR-218-5p inhibition in HOS/DXR and U2OS/DXR cells (Figure 7a and b). Therefore, we demonstrated that circSAMD4A positively regulated KLF8 via miR-218-5p in osteosarcoma cells.

### 3.8 CircSAMD4A knockdown enhances the cytotoxicity of DXR in osteosarcoma *in vivo*

**Figure 8:**
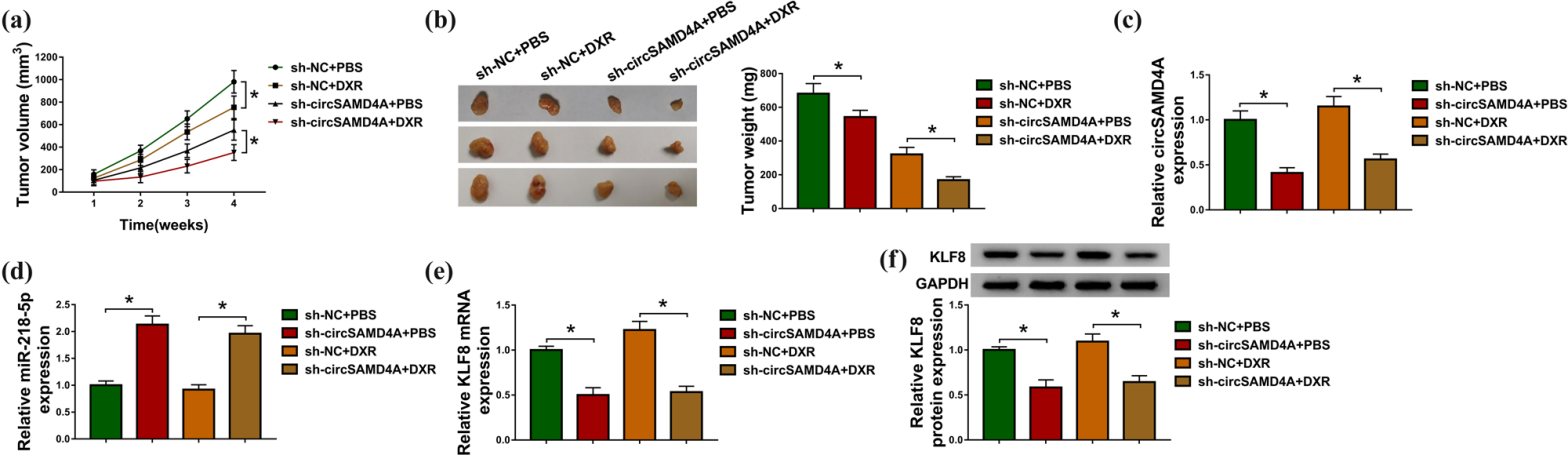
CircSAMD4A knockdown enhances the cytotoxicity of DXR in osteosarcoma *in vivo*. (a) Tumor volumes were calculated every week. (b) Tumor masses were collected and weighed on day 28. (c–e) qRT-PCR analysis of circSAMD4A, miR-218-5p and KLF8 expression in the tumor masses of each group. (f) Western blot analysis of KLF8 protein in tumor masses of each group. *P < 0.05.

The function of circSAMD4A in DXR-induced tumor growth suppression *in vivo* was investigated. The results suggested that circSAMD4A silencing accelerated DXR-induced suppression of tumor growth *in vivo* (Figure 8a and b). Afterward, molecular analysis exhibited that sh-circSAMD4A injection successfully induced the downregulation of circSAMD4A levels in the tumor masses (Figure 8c). Besides, circSAMD4A silencing increased miR-218-5p and decreased KLF8 expression *in vivo* (Figure 8d–f). Thus, we concluded that circSAMD4A knockdown promoted DXR-induced tumor suppression in osteosarcoma murine xenograft models by regulating miR-218-5p and KLF8 expression.

## 4 Discussion

Osteosarcoma is a highly aggressive bone sarcoma, and with the advances in complete surgical resection and multi-agent chemotherapy, up to 70% of patients with localized limb tumors and high-grade osteosarcoma become long-term survivors [22]. Nevertheless, osteosarcoma patients with nonresectable, primary metastatic or relapse tumors still have poor prognosis [23]. The acquisition of drug resistance accounts for the majority of poor effects of chemotherapy in osteosarcoma. DXR is one of the most effective drugs for osteosarcoma standard chemotherapy, and a total of 40–45% of high-grade osteosarcoma patients are unresponsive or only partially responsive to DXR [24].

Tumor chemoresistance is a complex, multistep process hallmarked by a number of abnormal genes, proteins, ncRNAs and some related signal pathways [25]. Increasing research studies have indicated that circRNAs are critical mediators in DXR resistance in many cancers. For example, Liang et al. revealed that circKDM4C suppressed cell proliferation, metastasis and DXR resistance in breast cancer by regulating PBLD via miR-548p [26]. Shang’s team identified 49 circRNAs that were differentially expressed in DXR-resistant and sensitive THP-1 acute myeloid leukemia cell lines, and among them, circPAN3 knockdown could sensitize resistant THP-1 cells to DXR through the miR-153-5p/miR-183-5p-XIAP axis [27]. Importantly, Kun et al. demonstrated that circPVT1 knockdown restored DXR and cisplatin sensitivity of osteosarcoma cells via reducing ABCB1 expression [9]. Thus, targeting circRNAs may be a promising therapeutic strategy for DXR resistance. In this study, we found that circSAMD4A was elevated in osteosarcoma, especially in DXR-resistant cell lines and tissues. Then we demonstrated that circSAMD4A knockdown rescued the DXR sensitivity of DXR-resistant osteosarcoma cells by mediating the suppression of cell viability, migration and invasion, as well as the arrest of the cell cycle *in vitro*. Besides, murine xenograft models indicated that circSAMD4A silencing enhanced the cytotoxicity of DXR in osteosarcoma *in vivo.* Thus, circSAMD4A knockdown inhibited DXR resistance in osteosarcoma.

It has been reported that circRNAs contain diverse types and quantities of miRNA binding sites and can act as miRNA sponges, regulators of splicing and transcription and modifiers of parental gene expression, thereby they are implicated in a series of biological and pathological processes as well as drug resistance [28,29]. To verify whether circSAMD4A could act as a miRNA sponge, bioinformatics analysis was used. Among the predicted candidates, miR-218-5p was selected for further exploration owing to the anticancer effects of miR-218 in osteosarcoma [30,31,32]. As for miR-218-5p, it was reported to regulate tumor cells’ malignant biological behavior in a series of human cancers, such as non-small cell lung cancer and triple negative breast cancer [15,16]. In the present research, we first validated that circSAMD4A sequestered miR-218-5p through functioning as a miR-218-5p sponge and negatively regulated its expression. MiR-218-5p was decreased in DXR-resistant osteosarcoma tissues and cells, and miR-218-5p inhibition enhanced the viability, migration and invasion but induced cell cycle arrest in DXR-resistant osteosarcoma cells. More importantly, inhibition of miR-218-5p reversed the restoration of DXR sensitivity induced by circSAMD4A silencing in osteosarcoma.

Subsequently, we further identified the molecular targets of miR-218-5p using online software TargetScan. KLF8 harbored a putative complementary sequence for miR-218-5p. KLF8 is a dual transcription factor and can either suppress or activate the transcription of target genes, including cyclin D1, KLF4 and E-cadherin [33], which are related to tumor development in diverse cancer types including osteosarcoma [20,21]. Additionally, recent studies also exhibited that KLF8 contributed to chemoresistance in breast cancer [19], gastric cancer [33] and glioma [34]. In this study, we uncovered that KLF8 was elevated in DXR-resistant cell lines and tissues, and KLF8 knockdown promoted the cytotoxicity of DXR in osteosarcoma. Importantly, this study first verified that miR-218-5p directly targeted KLF8, and miR-218-5p regulated DXR resistance via KLF8 in osteosarcoma. In addition, we also revealed that circSAMD4A positively regulated KLF8 through acting as a sponge of miR-218-5p, and thus, a circSAMD4A/miR-218-5p/KLF8 network in osteosarcoma cells was identified.

In conclusion, this study demonstrated that knockdown of circSAMD4A or KLF8 and elevation of miR-218-5p restored the DXR sensitivity of osteosarcoma cells. Besides, we also found that the function of circSAMD4A in osteosarcoma was partially exerted via the miR-218-5p/KLF8 axis, which provided new insight into the mechanisms underlying the chemoresistance of osteosarcoma and potential therapeutic targets for osteosarcoma chemotherapy.
